# Synthesis of naphthalimide-type chemsensor and its application in quality evaluation for *polygonatum sibiricum* Red

**DOI:** 10.3389/fchem.2022.969014

**Published:** 2022-08-11

**Authors:** Zhen Wang, Qiaoxu Sun, Yuanyuan Zhao, Jiao Du, Bin Wang

**Affiliations:** Key Laboratory of Xin’an Medicine of the Ministry of Education and Institute of Pharmaceutical Chemistry, Anhui University of Chinese Medicine, Hefei, China

**Keywords:** quality evaluation, detecting monosaccharides, HPLC analysis, 4-hydrazino-1,8-naphthalimide, polygonatum sibiricum polysaccharides

## Abstract

The premise and key of ensuring the safety and effectiveness of traditional Chinese medicine (TCM) is to construct appropriate quality evaluation system of TCM. This study aimed to establish a pre-column derivatization HPLC method for achieving the quality control of *Polygonatum sibiricum* by reacting synthesized 4-hydrazino-1,8-naphthalimide (HAN) with diverse monosaccharides from the hydrolytic product of *P. sibiricum* polysaccharides (PSPs), followed by HPLC separation. The HAN was synthesized based on a CuI-catalyzed cross-coupling reaction in water, and then employed as a novel chemosensor that reacts with reducing sugars. Good separation was achieved at a detection wavelength of 448 nm using an ZORBAX SB-C8 column under a gradient elution at a flow rate of 0.5 ml/min within 12 min. The monosaccharide compositions of PSP mainly include two hexoses [glucose (Glc), galactose (Gal)] and two hexuronic acids [glucuronic acid (GlcA) and galacturonic acid (GalA)], and the molar ratio of Glc, Gal, GlcA and GalA is 16.67:52.94:10.58:19.81. The verified HPLC method, possessing excellent precision and good accuracy, successfully achieved rapid qualitative and quantitative determination for PSP*.* Additionally, the HAN displayed fluorescence enhancement through “push–pull” mode, and fluorescence decreased through “pull–pull” mode after binding to monosaccharides, which is a potential for fluorescence determination of different monosaccharides.

## Introduction

Polysaccharides are extremely important pharmacological active components exist in most TCM, for instance, *Polygonatum* species (Zhao et al., 2018; Liu et al., 2022)*.* The main and active components, TCM *Polygonatum* polysaccharides, exhibited antioxidant activity (Zhang et al., 2019), anti-osteoporosis (Yelithao et al., 2019), immunomodulatory effects (Zhao et al., 2019), as well as preventing depression-like behaviors (Shen et al., 2021). However, the intrinsic polydispersity and lack of chromophore of polysaccharides makes the quality control for TCM polysaccharides greatly difficult to achieve. Furthermore, in the herbal market, the complex sources of TCM increase the latent risk of ineffective in clinical practice. These facts make the quality evaluation for TCM polysaccharides extremely important.

Carbohydrates are essential to life which are found everywhere in nature ranging from simple monosaccharides to complex oligosaccharides, as well as polysaccharides (Williams et al., 2021). However, saccharides cannot be detected by ultraviolet (UV) and fluorescence detector due to the absence of UV absorption and fluorescent groups. The content of total saccharides in crude polysaccharides extract mainly employs the colorimetric method after acidification to determine (Su and Li, 2020). However, the sensitivity and accuracy are low for polysaccharides, and even lower when the polysaccharides possess relatively low purity, for instance, the polysaccharides samples combining a certain amount of pigment, nucleic acid and protein, respectively.

Currently, two approaches, direct detection and chemical derivation, were adopted for measuring monosaccharides. The direct detection method mainly takes advantage of high-performance liquid chromatography combined evaporative light scattering detector (Sharma et al., 2010) or pulsed amperometric detector (Gangola et al., 2014). Low detection limits and requiring expensive columns exist in the method. Additionally, chemical derivations of monosaccharides method mainly make use of gas chromatography coupled with mass detector (Xia et al., 2018), and liquid chromatography combined with highly sensitive detector, such as ultraviolet, fluorescence and mass spectrometer (Lloyd and Doherty, 1952; Pauk et al., 2017). Chemical derivatization, especially pre-column derivatization which only need different derivatization reagents, such as 1-phenyl-3-methyl-5-pyrazolinone (PMP) (Zhao et al., 2020), fluorescein isothiocyanate (FITC) (Zhang et al., 2018), aminophenamide (2-AB) (Adamczyk et al., 2014) and 8-aminophenyl 1, 3, 6-trisulfonic acid (ANTS) (Deng et al., 2018), to reaction with monosaccharides, greatly improves measured sensitivity and selectivity.

With the help of high-resolution liquid chromatography, the pre-column derivatization strategy help achieving a series of successes for the quality control for TCM polysaccharides (Dai et al., 2010). Three major *Polygonatum* species, namely *Polygonatum kingianum* Coll. Et Hemsl. (Li et al., 2020), *Polygonatum Sibiricum* Red. (Wang et al., 2020), and *Polygonatum cyrtonema* Hua (Zhang J. Y. et al., 2021), are officially recorded in Chinese pharmacopoeia. Due to the higher medicinal effect in clinical, the *P. Sibiricum* Red. was widely used to construct prescription (Mu et al., 2021). Polysaccharides, accounting for more than 20% of the total components, regarded as the representative Q-markers to evaluate the quality of *Polygonum* species (Liu and Si, 2018). Based on our previous practice in the quality evaluation for *Angelicae pubescentis* radix (Wang et al., 2014), Wu-Wei-Wen-Tong Capsule (Jiang et al., 2021), Nao-Luo-Xin-Tong (Wang et al., 2019), especially for PSP by common pre-column derivatization PMP-HPLC approach (Shen et al., 2021), we herein developed a novel HAN-HPLC strategy, successfully achieving rapid qualitative and quantitative determination for PSP. Higher sensitivity (*ɛ* = 12882 for PMP; *ɛ* = 16138 for HAN) and less noise interference (detection at 245 nm for PMP and 448 nm for HAN) of chemsensor HAN and followed by good separation for derived monosaccharides acquired by HPLC technique was also achieved, highlighting the merit of novel strategy.

## Materials and methods

### Reagents and instrument

The rhizomes of *Polygonatum Sibiricum* Red. (PS) were picked from the Banzhuyuan in Jinzhai County (Anhui province, China). Monosaccharide standards including arabinose, glucose, glucuronic acid, galactose, galacturonic.

Acid, mannose, rhamnose, ribose, and xylose (purity >97%) were purchased from Shanghai Bide Medical Technology Co., LTD. We obtained 1-phenyl-3-methyl-5-pyrazolone (PMP) from the Maclean Biotechnology Co., Ltd. ^1^H NMR and ^13^C NMR spectra were recorded on an AV-600 spectrometer and the analytes were dissolved in DMSO-*d*_6_ solution. The chemical shifts (*δ*) value is expressed in ppm relative to TMS (0.00 ppm) and coupling constants (*J*) in Hz for ^1^H NMR and ^13^C NMR. High-Resolution Mass spectra (HRMS) data were obtained from a Waters Xevo G2-XS QTOF spectrometer (Tolerance = 10.0 ppm). Ultraviolet-visible Absorption spectra were recorded using a SHIMADZU UV-2550 spectrophotometer. Fluorescence measurements were performed on a SHIMADZU RF-5301 fluorescence spectrometer at room temperature.

### Synthesis of naphthalimide-type chemsensor

For the synthesis of naphthalic anhydride hydrazine hydrochloride salt (NAHC), a previous method was adopted (Kumar and Ma, 2018). A 10 ml resealable screwcap Schlenk tube equipped with a Teflon-coated magnetic stir bar was charged with CuI (9 mg, 8 mol%), BMPO (14 mg, 8 mol%), CTAB (25 mg, 16 mol%), 4-Bromo-1,8-naphthalic Anhydride (169 mg, 0.6 mmol), K_3_PO_4_ (25 mg, 0.12 mmol, 0.2 equiv.), H_2_O (0.6 ml) and the resulting mixture were stirred at 80°C for 15 min, then K_3_PO_4_ (127 mg, 0.6 mmol, 1.0 equiv.) and hydrazine hydrate (30 μL, 0.6 mmol) were added. The Nitrogen gas was bubbled through the reaction mixture for 15 min, then stirred in a closed test tube at 80°C for 8 h until the starting material was consumed totally. After cooling to room temperature, the reaction mixture was diluted with dichloromethane, and then filtered. The filtrate was washed with brine, and then the organic layer was separated and acidified to pH = 3-4 by adding 37% HCl solution. The resulted precipitate was filtered, washed with dichloromethane and dried at room temperature to afford the corresponding naphthalic anhydride hydrazine hydrochloride salt (NAHC, 64 mg, yield: 38.6%). ^1^H NMR (500 MHz, DMSO-*d*_6_) δ 8.59 (d, *J* = 7.3 Hz, 1H), 8.56—8.52 (m, 1H), 8.35 (d, *J* = 7.9 Hz, 1H), 8.23 (d, *J* = 7.8 Hz, 1H), 8.05—7.97 (m, 1H). ^13^C NMR (126 MHz, DMSO-*d*_6_) δ 160.5, 133.3, 132.2, 131.9, 131.5, 130.2, 129.9, 129.4, 127.5, 122.8, 122.1.

For the synthesis of 4-hydrazino-1,8-naphthalimide (HAN) (Ye et al., 2014), a 10 ml resealable screwcap Schlenk tube equipped with a Teflon-coated magnetic stir bar was charged with NAHC (132 mg, 0.5 mmol), n-butylamine (300 μL, 3.0 mmol), ethanol (3 ml) and the resulting mixture were stirred at 85°C for 6 h until the starting material was consumed totally. After cooling to room temperature, the reaction mixture was washed with saturated sodium bicarbonate, and extracted with dichloromethane. The organic layer was washed with brine, and then dried over Na_2_SO_4_ and concentrated in vacuo. The residue was purified by column chromatography on silica (petroleum ether/ethyl acetate = 1/2) to afford the HAN (33 mg, yield: 25%). ^1^H NMR (400 MHz, DMSO-*d*_6_) δ 8.75 (dd, *J* = 8.5, 1.2 Hz, 1H), 8.47 (dd, *J* = 7.3, 1.0 Hz, 1H), 8.29 (d, *J* = 8.6 Hz, 1H), 7.87 (s, 1H), 7.72 (dd, *J* = 8.4, 7.3 Hz, 1H), 6.80 (d, *J* = 8.7 Hz, 1H), 1.72 (s, 2H), 1.51—1.40 (m, 2H), 0.98 (t, *J* = 7.4 Hz, 3H). ^13^C NMR (126 MHz, DMSO-*d*_6_) δ 133.54, 132.38, 132.13, 131.76, 130.07, 129.58, 39.04, 29.59, 19.77, 14.10.

### Derivatization reaction

The reaction principle based on hydrazine group and carbonyl group from monosaccharide molecule has been reported by different research groups (Lattova and Perreault, 2003; Iqbal and Novalin, 2009; Han et al., 2016). Here, the synthesized HAN compound that has a hydrazine group in the molecule skeleton reacted with monosaccharide to successfully yield the conjugate HAN-monosaccharides. Briefly, a 10 ml Schlenk tube equipped with a Teflon-coated magnetic stir bar was charged with HAN (50 μmol), monosaccharide (50 μmol), acetic acid (5 μL, 2 eq), ethanol (0.5 ml) and the resulting mixture was stirred at 80°C for 4 h until the starting substances disappear thoroughly. A new point, namely HAN-monosaccharides, was formed with a Rf = 0.5 (CH_2_Cl_2_:CH_3_OH = 5:1). After cooling to room temperature, the reaction mixture was diluted with H_2_O, and extracted with dichloromethane. The water layer was concentrated in vacuo and then filtered, finally diluted with CH_3_OH prior to HPLC analysis.

### Optimizing derivatization condition

The setting of reaction conditions has great influence on the reaction results. Therefore, the choice of reaction parameters is significantly important. Currently, response surface methodology (RSM) is widely used to screen out the optimum result (Yuan et al., 2020). We herein selected five single factors including time (h), reaction temperature (°C), kinds of acid, acid concentration (eq) and molar Ratio (eq) for optimizing the efficiency of derivatization of saccharides. The results of single factor experiment were shown in Supplementary Table S1. Box–Behnken design (BBD)-RSM were used to investigate the yield of HAN-monosaccharide (Supplementary Table S1). The parameters of the model were estimated by the least square method on the basis of 46 measurement experiments, and then the model is established (Supplementary Table S2). By applying multiple regression analysis to the experiment data, the response variable and the test variables were related by the second-order polynomial equation. The obtained test data were used for multiple regression fitting and optimization of process parameters by using Design-Expert 11.0 software. The best work conditions were used for verification and detection, compared with the ideal value of the software.

### Investigation of spectroscopic characteristics

The spectral properties of the derivatization reagent and its conjugated products with monosaccharides were further investigated. The solution of compound HAN was prepared in CH_3_OH (1.76 mM). 0.4 ml HAN solution was diluted in 9.6 ml CH_3_OH (0.07 mM) for ultraviolet measurements. 0.1 ml HAN, and PMP solutions were diluted in 9.9 ml CH_3_OH (0.02 mM) for molar absorptivity measurements. Using pure methanol solution as a blank, full-wavelength scanning, the absorbance value under the maximum absorption wavelength was recorded, and the operation was repeated three times. For fluorescence measurements, the test solutions of HAN-monosaccharides (5 μM) were prepared with excitation set at 448 nm, and the excitation and emission slit widths both were 10 nm (Sun et al., 2019). For Fourier transform infrared (FT-IR) analyses, HAN-Glc (2 mg) was mixed with dried potassium bromide (200 mg) to ground and pressed under a vacuum condition by using a Nicolet 5700 FT-IR spectrometer and recorded in the wavelength region of 4,000–400 cm^−1^.

### High performance liquid chromatography

The experiments were operated on an Agilent 1,260 series Liquid Chromatography coupled with UV-visible detector. A ZORBAX SB-C8 (250 mm × 4.6 mm, partical size 5 μm). Mobile phase consisted of ultrapure water-methanol (40:60, v/v). The flow rate was 0.5 ml/min. Column temperature and detector temperature kept at 25°C. Detection wavelength was set at 448 nm. The injection amount was 10 μL.

### Preparation of *p. sibiricum* polysaccharide

PSP were obtained from *P. sibiricum* powder using water soaking (1:4 w/v, 100°C) with boiling 30 min, followed by 4 times volumes ethanol precipitation. The precipitates were then deproteinized by the Sevag’s method and the purified PSPs after lyophilization were collected for further analysis (Wang et al., 2020; Shen et al., 2021).

### Monosaccharide determination

First, PSPs was hydrolyzed into monosaccharides with 2 M trifluoroacetic acid, and then derivatized with HAN. After extraction with dichloromethane, the aqueous solution of saccharides derivatization was obtained, and then detected by optimum HPLC method after appropriate dilution. Finally, the monosaccharide kinds and composition in PSP were analyzed, and a novel quality control method for *P. Sibiricum* Red. was established.

## Results and Discussion

### Synthesis of HAN- monosaccharides

**FIGURE 1:**
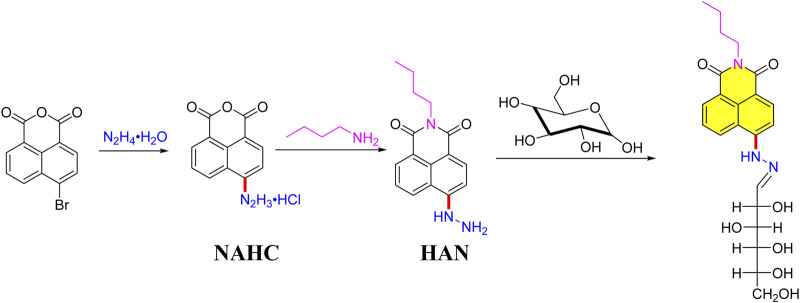
The derivatization reaction and its possible reaction mechanism of HAN-Glc.

As shown in Figure 1, the pre-column derivatization HAN was prepared starting from the 4-bromo-1,8-naphthalimide reacts with hydrazine hydrate, providing NAHC under 37% HCl solution. Next, with the help of n-butylamine, desired HAN was obtained by 25% yield. Based on the optimum derivatization condition under *Optimizing derivatization condition* Section, the targeted HAN-Glc was successfully obtained in solvent ethanol.

### Derivatization condition by response surface methodology

To improve the separation efficiency and HPLC detection sensitivity, 4-hydrazino-1,8-naphthalimide (HAN) was used in this study for derivatization of monosaccharides. After derivatization, the introduction of fluorescent group can improve detection efficiencies of saccharides.

**FIGURE 2:**
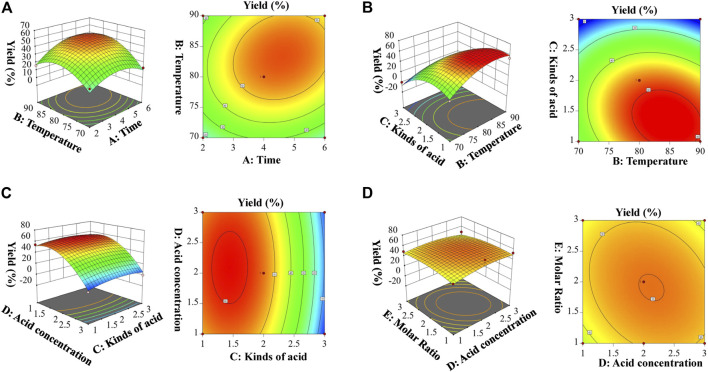
Response surface plots showing effects of variables on the derivatization of HAN. [**(A)**. The response surface of the effect of exaction time (A, h) and temperature (B, °C); **(B)**. The response surface of the effect of temperature (B, °C) and kinds of acid (C); **(C)**. The response surface of the effect of kinds of acid (C) and acid concentration (D, eq); **(D)**. The response surface of the effect of acid concentration (D, eq) and molar Ratio (E, eq)].

Based on single factor investigation results, 46 runs of Box–Behnken test factors design, and results of test factors are shown in Supplementary Table S2. The results showed that the yield of HAN-monosaccharide varied from 0 to 62. The quadratic regression equation with the yield of HAN-monosaccharide as the objective function was obtained. The F-test and *p*-values were used to measure the significance of the coefficients of the model. As shown in Supplementary Table S3, A (Time) were significant (*p* = 0.0018); B (Temperature) and C (Kinds of acid) was highly significant (*p* < 0.0001). These data indicate that the model established by the experiment was feasible. The precision value of 16.129 indicates that the model can predict experimental results. An R^2^Adj value of 0.8961 indicates that the model can prove the prediction of 89.61% of the response value, and the determination coefficient *R* ^2^ of 0.9423 indicates that the model has a good degree of fit, and the yield value of the HAN-monosaccharide can be analyzed and predicted. The R^2^Pred being equal to 0.7899 is not significantly different from the *R* ^2^ of 0.9423, indicating that there was no need to further optimize the response surface equation. Design-Expert (version 11.0) was used to create the relationship between the independent and dependent variables, and the 3D response surface and contour plots are shown in Figure 2. Figure 2A displayed the response surface and contour map of A (Time) and B (Temperature) to the yield of HAN-monosaccharide. The intensity of contour in map and the steepness of response surface can be used to analyze the influence degree of interlacing factors on response surface. The greater the density and slope of the response surface, the greater the impact degree. Obviously, the map of A and B is the steepest (corresponding Figures 2A,B, respectively), and the map of D and E is flattest (corresponding Figures 2C,D, respectively). These results illustrated that time (A) and temperature (B) have an important impact on the yields of HAN-monosaccharide, but acid concentration (D) and molar ratio (E) have a poor impact. So, in this experiment, the derivation conditions of HAN-monosaccharide were optimized by using RSM, and the optimization conditions for derivatization of monosaccharide were as follows: Time: 4 h, Temperature: 80°C, Kinds of acid: acetic acid, Acid Concentration: 2 eq, and Molar ratio: 1:1.

### UV-vis spectra of HAN

**FIGURE 3:**
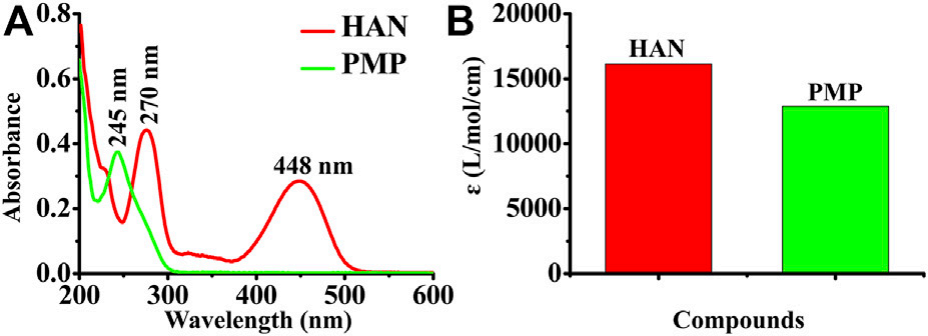
**(A)** UV on the derivatization of naphthalimide hydrazine; **(B)** The ɛ value of HAN and PMP in CH_3_OH.

Based on Lambert-Beer law (A = lg (1/T) = *ε*bc), the molar extinction coefficient of synthesized HAN could be obtained by UV-visible spectrophotometer. According to the Lambert-Beer law, the molar extinction coefficient was calculated. The result showed that the main ultraviolet absorption peak of PMP is at 245 nm, and two absorption peaks of HAN are at 277 and 448 nm respectively, which are larger than PMP (Figure 3A). The absorption peak at 448 nm indicates that HAN has fluorescence absorption. By calculating the molar absorption coefficient in CH_3_OH, *ε* (PMP) = 12881.92 L/mol/cm, *ε* (HAN) = 16138.41 L/mol/cm, it indicates that HAN possess highly sensitive than PMP (Figure 3B).

### Mechanism analysis of HAN-glc by FT-IR, UV-vis, NMR, and HRMS

The possible derivatization mechanism was further investigated through a series of physical devices including FL spectrophotometer, FT-IR spectrophotometer, UV-Vis and NMR technique, as well as HRMS.

**FIGURE 4:**
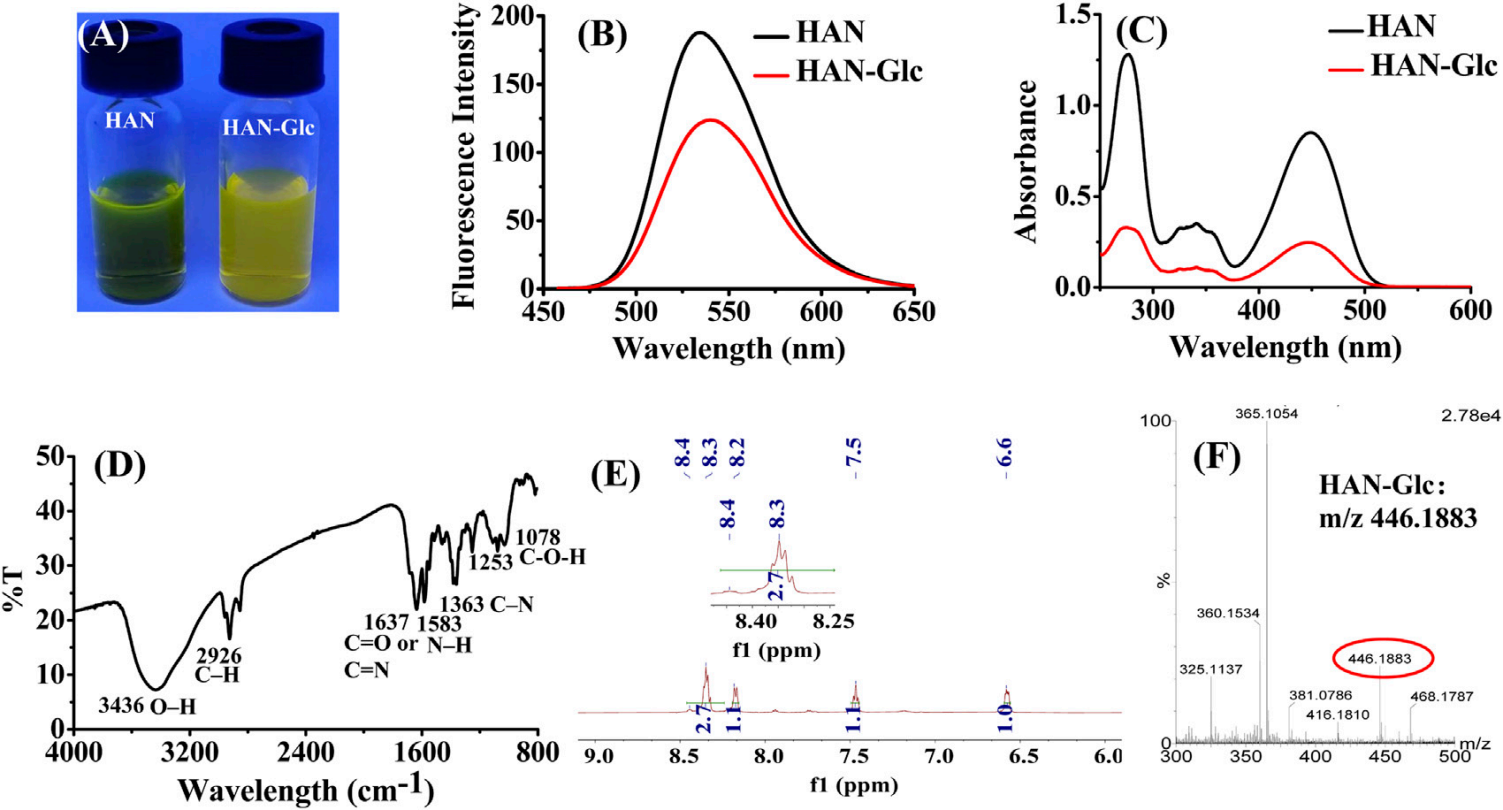
Mechanism analysis: **(A)** Fluorescence photograph of HAN and HAN-Glc solutions under a wavelength of 365 nm; **(B)** fluorescence spectrum of HAN and HAN-Glc; **(C)** UV-Vis spectrum of HAN and HAN-Glc, **(D)** FT-IR profile of HAN-Glc, **(E)** NMR spectra of HAN-Glc, and **(F)** HRMS data of HAN-Glc.

As the picture being shown in Figure 4A, the methanol solution of HAN showed strong fluorescence under 365 nm fluorescent lamp. However, the conjugate HAN-Glc display weak yellow fluorescence. This naked-eye observation result is consistent with fluorescence spectrometry detection presented in Figure 4B. Compared with HAN, the ultraviolet absorption spectrum of the conjugate is slightly weakened, but the main absorption peak (at 277 and 448 nm) still remains (Figure 4C), which provided a possibility for further HPLC-UV analysis.

To further investigate the molecular structure characteristics of the conjugate HAN-Glc, infrared spectroscopy and NMR analysis were adopted. As the FT-IR spectra of conjugate HAN-Glc being shown in Figure 4D, the stretching vibration of O–H or C–H residue exhibited the peak at 3,436 cm^−1^, and the C–H stretching in Glc appeared at 2,926 cm^−1^ (Dong et al., 2021). The absorption bands at 1,637 cm^−1^ were related to the C=O or C=N stretching vibration (Demircioglu et al., 2014; Zhu et al., 2021). The peak at 1,363 cm^−1^ assigned to the stretching of C–N, and the peak at 1,583 cm^−1^ was attributed to the N–H bending in HAN-Glc (Zhang S. et al., 2021). Moreover, the absorption peaks at 1,253 and 1,078 cm^−1^ were assigned to the C-O-H link bonds (Wang et al., 2021). The above FT-IR result displayed a strong C=N double bonds was formed, demonstrating that glucose molecule bonded with the derivatization agent HAN and producing desired HAN-Glc. In addition, the formation of C = N double bonds was also observed in NMR spectra, and corresponding ^1^H NMR data of crude conjugate HAN-Glc were showed as follow: ^1^H NMR (600 MHz, Methanol-*d*_4_) δ 8.34 (d, *J* = 7.1 Hz, 3H), 8.18 (d, *J* = 8.7 Hz, 1H), 7.47 (s, 1H), 6.59 (s, 1H), 4.83 (s, 32H), 4.76 (d, *J* = 5.2 Hz, 1H), 4.53 (d, *J* = 18.8 Hz, 1H), 4.37 (dd, *J* = 57.8, 8.3 Hz, 1H), 3.85—3.74 (m, 1H), 3.75—3.66 (m, 2H), 3.64 (td, *J* = 9.6, 9.1, 6.1 Hz, 1H), 3.60 (s, 1H), 3.54—3.37 (m, 2H), 3.26 (s, 5H), 1.74—1.67 (m, 2H), 1.46 (q, *J* = 7.5 Hz, 2H), 1.22 (s, 11H), 0.98 (t, *J* = 7.4 Hz, 3H), 0.84 (s, 3H). It is worth noting that the δ = 8.34 was presented in ^1^H NMR of HAN-Glc (see Figure 4E). As well known, the chemical shift of hydrogen located at the carbon of C=N bond generally below δ = 8.4 (Owen et al., 2009). Therefore, we infer that the analyte contains C=N double bonds in its structure. In addition, HRMS spectra also provides further support. As shown in Figure 4F, the peak at m/z 446.1883, which was assignable to [HAN-Glc + H]^+^ (calc. m/z 446.1883) in the ESI mass spectrum.

### Method validation

A series of experiments regarding linearity, LOD, LOQ and reproducibility were performed to validate the developed method. For the construction of the calibration curves, different concentrations of Glc, Man, Rha, Gal, GlcA, GalA standards in the range of 45–776 μg/ml. The calibration curves were constructed by plotting the peak area against the spiked concentrations of Glc, Man, Rha, Gal, GlcA, GalA, and conducting a linear fitting to the result. The linear regression, LOD and LOQ data are listed in Table 1. As shown in Table 1, satisfactory correlation coefficients for the six compounds were obtained ranging from 0.9990 to 0.9993. The LOD values for the six sugars were in the range of 10.82–76.09 μg/ml. The precision of the proposed method was repeatedly tested after 8 times of continuous injection, based on which the relative standard deviation (RSD) of 6 mixtures of monosaccharides were calculated. The RSDs were in the range of 0.78–1.34% (Table 2). The stability of the proposed method was evaluated by being injected 5 times at 4 h and once 24 h after mixing. The results showed that the RSDs were in the range of 0.80–2.93% (Table 2), indicating that the proposed method provides good reproducibility. The recoveries at three different concentrations of 6 kinds of monosaccharide were mixed with standard solutions in the linear range for carrying out the sample adding recovery test. Each sample was repeatedly injected 3 times. The average value for each component were obtained by comparing the amounts calculated from the calibration curves with the corresponding spiking amounts. As shown in Table 2, the recoveries were in the range of 99.22–103.70%, demonstrating a satisfactory accuracy of the proposed method. The validated method possesses well linear relation, good accuracy, as well as high sensitivity and stability, and thus could meet the requirement of determination of Glc, Man, Rha, Gal, GlcA, GalA in polysaccharides.

**TABLE 1:** Linearity, regression line and LOD of the derivatives with the proposed method.

Monosaccharides	T_R_ (min)	Linear equation	Linear range (μg/mL)	*R* ^2^	LOD (μg/mL)	LOQ ((μg/ml)
Glc	11.71	y = 8576.8x + 319.13	45–540	0.9992	23.49	165.12
Rha	16.31	y = 3,864.1x + 194.23	82–492	0.9993	54.88	300.22
Man	12.76	y = 2,975.8x - 22.823	45–72	0.9993	10.82	18.18
Gal	10.77	y = 1,242.6x - 41.423	45–72	0.9990	40.27	56.46
GlcA	4.03	y = 467.37x + 36.341	194–776	0.9993	57.24	372.24
GalA	4.92	y = 528.3x - 24.443	97–776	0.9990	76.09	145.67

**TABLE 2:** Precisions, Stability test, Repeatability test and Sample adding recovery test of N2-monosaccharides.

Monosaccharides	T_R_ (min)	Mean (μg/ml)	Precision	Stability	Repeatability	Sample adding recovery
			RSD (%)	RSD (%)	RSD (%)	(%)
Glc	11.71	90	0.94	2.12	4.41	101.44
Rha	16.31	82	1.34	2.93	3.33	102.25
Man	12.76	90	1.17	0.82	2.64	99.81
Gal	10.77	90	0.78	1.21	1.03	103.70
GlcA	4.03	97	0.81	0.80	0.61	99.22
GalA	4.92	97	0.79	0.73	0.57	100.36

### Quality control for *polygonatum sibiricum* red

**FIGURE 5:**
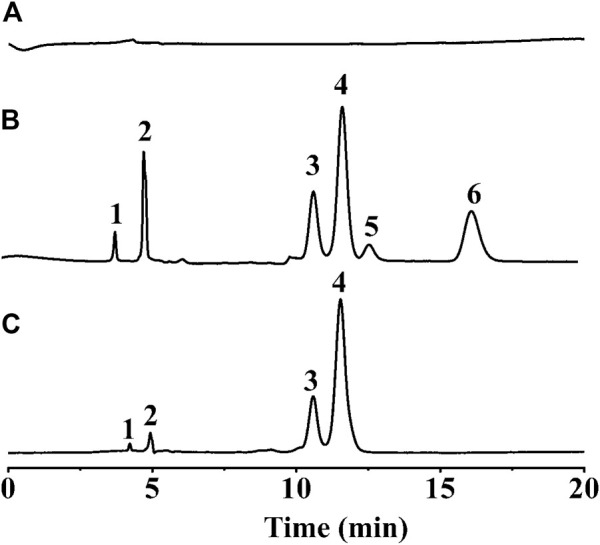
**(A)** HPLC-DAD chromatogram of mixing monosaccharide standard samples; **(B)** HPLC-DAD chromatogram of mixing HAN-monosaccharide standard samples; **(C)** HPLC-DAD chromatogram of PSP after derivatization; (Peak 1, HAN-GlcA, t_R_ = 4.03 min; Peak 2, HAN-GalA, t_R_ = 4.92 min; Peak 3, HAN-Gal, t_R_ = 10.77 min; Peak 4, HAN-Glc, t_R_ = 11.71 min; Peak 5, HAN-Man, t_R_ = 12.76 min; Peak 6, HAN-Rha, t_R_ = 16.31 min).

Indirect analysis of monosaccharides in PSP samples was performed by HPLC-DAD technique. In the established method, a HPLC column coupled with derivatization analytes was used for indirect analysis of saccharides. Due to lack of ultraviolet chromophores, monosaccharide molecules cannot be observed by ultraviolet technique. As displayed in Figure 5A, mixture containing six monosaccharides, were not observed in HPLC chromatogram using our established method. However, after derivatization reaction of HAN and each standard of six monosaccharides, Glc, Man, Rha, Gal, GlcA, and GalA, the metabolites of the different monosaccharide conjugated with HAN could be observed and even well separated using the established method, and corresponding results displayed in Figure 5B may meet the requirements of qualitative and quantitative detection. Subsequently, indirect analysis of monosaccharides in PSP samples was performed. The PSP samples were hydrolyzed and then derived using HAN. The obtained samples were tested by HPLC technique, and resulting HPLC chromatogram is shown in Figure 5C. As shown in Figure 5A, due to lack of ultraviolet chromophores, all monosaccharides cannot be observed under established HPLC-DAD chromatogram condition. Remarkably, according to the HPLC chromatogram of Figure 5B, we found that derivatives containing uronic acid, such as HAN-GlcA and HAN-GalA, generally with high polarity, peaked before 5 min. Subsequently, derivatives containing aldose, such as HAN-Gal, HAN-Glu, and HAN-Man, peaked between 10 min and 13 min. Finally, derivatives containing desose, HAN-Rha, with the lowest polarity, peaked at the end of the chromatogram. Because of the products HAN-monosaccharides possessing UV absorbance at 448 nm and thus could be observed by HPLC-DAD technique. The observed six peaks were identified and labeled: Peak 1, HAN-GlcA, *t*_R_ = 4.03 min; Peak 2, HAN-GalA, *t*_R_ = 4.92 min; Peak 3, HAN-Gal, *t*_R_ = 10.77 min; Peak 4, HAN-Glc, *t*_R_ = 11.71 min; Peak 5, HAN-Man, *t*_R_ = 12.76 min; Peak 6, HAN-Rha, *t*_R_ = 16.31 min. Therefore, this established method performed the well separation for HAN-monosaccharides, and by transformation, achieved the indirect determination for the content of different monosaccharides. The degree of separation of all six components aforementioned is greater than 1.5, ensuring the requirements for HPLC separation. Four peaks attributed to HAN-Glc, HAN-Gal, HAN-GlcA and HAN-GalA were found and the contents of HAN-Glc, HAN-Gal, HAN-GlcA and HAN-GalA are calculated according to the linear equation. The result was listed in Table 3. By transformation, the corresponding molar ratio of Glc, Gal, GlcA and GalA is 16.67:52.94:10.58:19.81 in PSP samples. The high content of galactose, different from other Polygonatum species such as *Polygonatum cyrtonema* Hua, is one of the main characteristics of *P. sibiricum* Red. The result is basically consistent with a previous report (Wang et al., 2020). Finally, we have successfully achieved the determination of monosaccharide composition in PSP analytes. Therefore, our constructed method can meet the quality control of *P. sibiricum* Red. polysaccharide.

**TABLE 3:** Determination of monosaccharide composition of PSP.

Monosaccharides	Concentration (μg/mg)	Molar %
Glc	60.55	16.67
Gal	192.27	52.94
GlcA	41.39	10.58
GalA	77.55	19.81

### Potential applications

**FIGURE 6:**
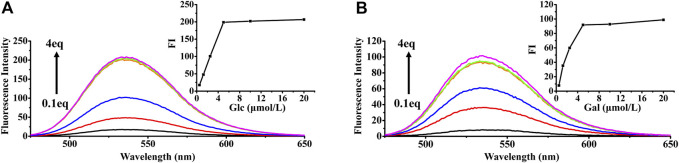
Fluorescence emission spectral (λex = 448 nm) of HAN (50 μmol) with increasing concentration of Glc **(A)** and Gal **(B)** in CH_3_OH. Inset: Plot of fluorescence intensity at 540 nm as a function of the HAN-Glc and HAN-Gal concentration.

The fluorescence properties of HAN were investigated in the presence of 9 monosaccharides (Glc, Xyl, Gal, Man, Ara, Rha and GalA) in CH_3_OH (Supplementary Figure S1). The derivatization of monosaccharides with HAN (1 equiv.) induced an obvious fluorescence enhancement at 448 nm. Among them, the fluorescence intensity of Man, Glc and Gal is the highest, Rha, GalA and GlcA is lower, Rib, Xyl and Ara (the equivalent is 2) is lowest. We inferred that the observed difference of fluorescence change mainly comes from three reasons: First, the formed imine bond by reacting HAN with various different monosaccharides be capable of rotation, resulting in the occurrence of internal charge transfer (ICT) and corresponding fluorescence signal decrease. Second, the transformation rate of different monosaccharides to corresponding HAN-monosaccharides are also diverse. The probe derivatives containing high concentrations were more severely quenched. Finally, molecular weight of different monosaccharides is partly responsible for the fluorescence change. Comparison with relatively smaller monosaccharides, the bigger saccharide molecule may slightly prevent molecular rotation at the position of formed imine part. Fluorescence detection results show that 6 kinds of monosaccharides including Man, Glc, Gal, Rha, GalA and GlcA could be used for the determination of derivatization. The concentration-dependent reaction of Glc and Gal with HAN along with the fluorescence signal change were also presented in Figure 6.

## Conclusion

In conclusion, we had developed a naphthalimide-based pre-column derivatization HPLC method, successfully achieving the quality control of *P. sibiricum* Red. polysaccharide. The synthesized HAN reacts with diverse monosaccharides under optimized conditions according to response surface methodology, and with the help of HPLC, we realized simultaneous quantitation of four monosaccharides from PSP. The synthesized HAN exhibited a turn-on fluorescence response toward monosaccharide with a bright orange fluorescence under UV radiation, implying that HAN is a potential for fluorescence determination of diverse monosaccharides.

## Data Availability

The original contributions presented in the study are included in the article/Supplementary Material, further inquiries can be directed to the corresponding author.
