# Supplementary material for: Synthesis of naphthalimide-type chemsensor and its application in quality evaluation for polygonatum sibiricum Red
Source: Front Chem. 2022 Aug 11;10:969014. doi: 10.3389/fchem.2022.969014 (PMC9402912; doi:10.3389/fchem.2022.969014)
Supplement: Supplementary file 1 [file DataSheet1.docx]

Supplementary Material

# Supplementary Figures and Tables

## Supplementary Figures


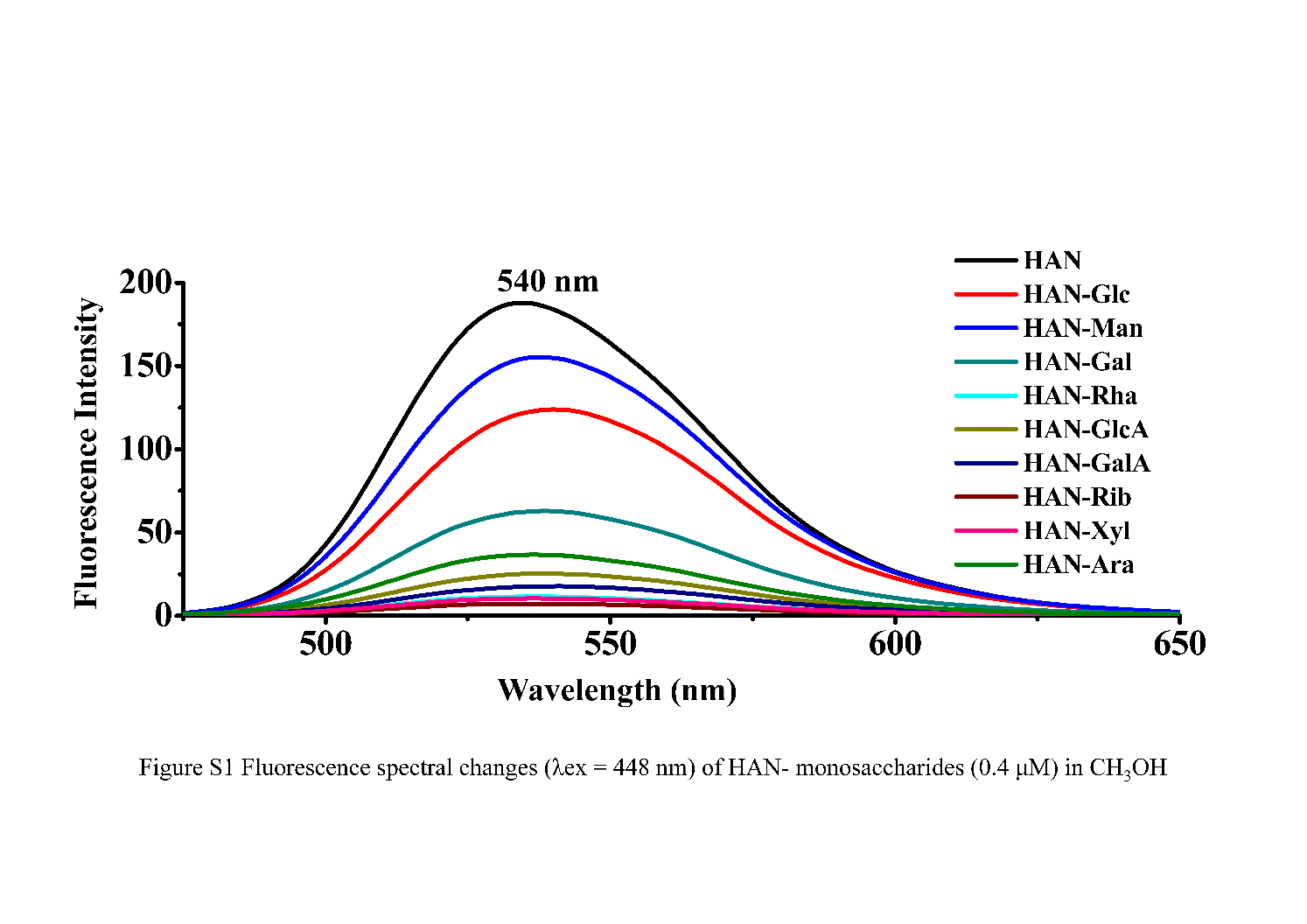


**Supplementary Figure 1.** Fluorescence spectral changes (λex = 448 nm) of HAN- monosaccharides (0.4 μM) in CH_3_OH.

**
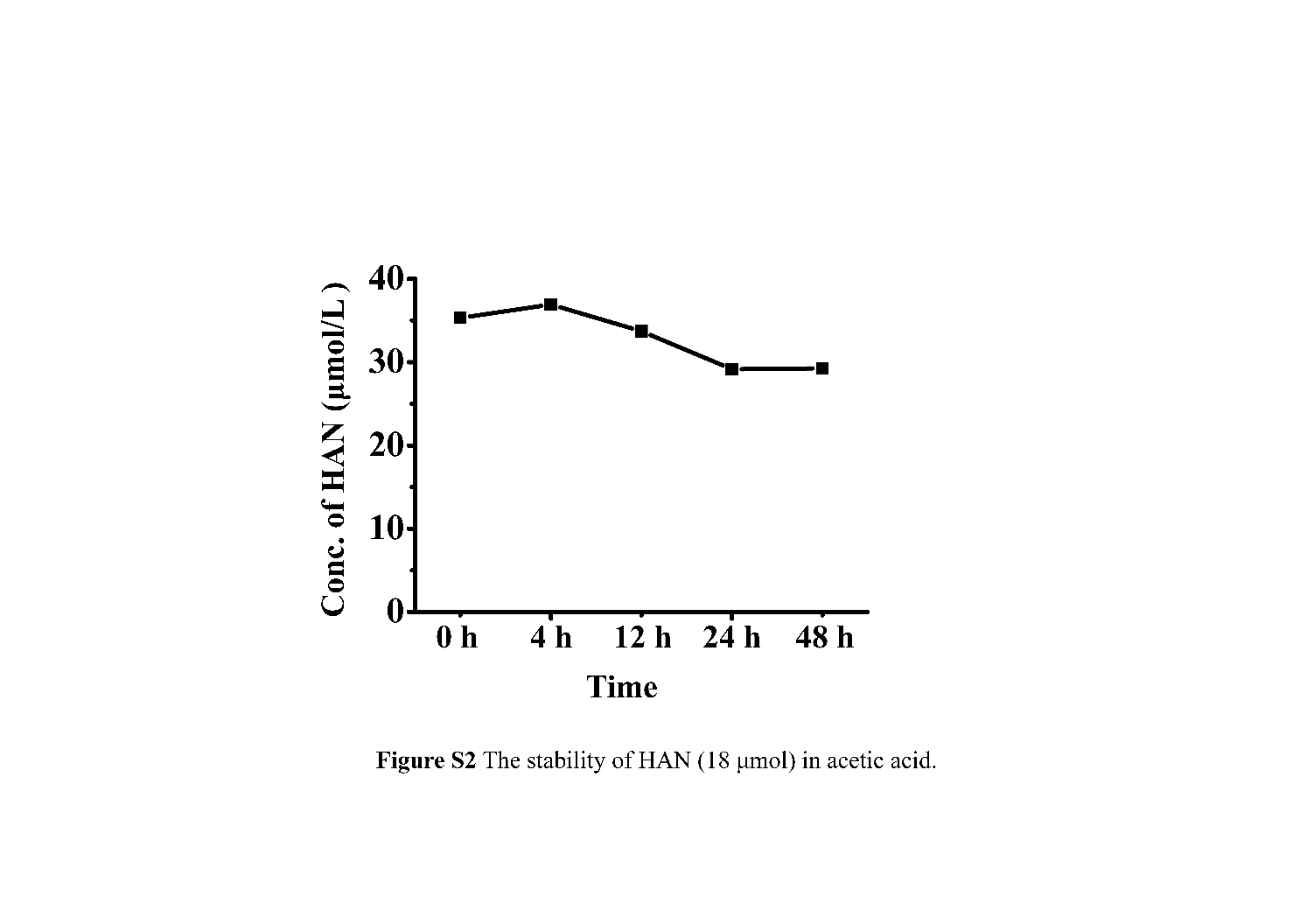
**

**Supplementary Figure 2** The stability of HAN exposed acetic acid in ethanol.

## 1.2 Supplementary Tables

**Supplementary Table 1** Levels and code of derivatization variables used in Box–Behnken design.

| Variable | Coded Levels | | |
| --- | --- | --- | --- |
|  | -1 | 0 | 1 |
| Time (A, h) | 2 | 4 | 6 |
| Temperature (B, ℃) | 70 | 80 | 90 |
| Kinds of acid (C) | Methanoic acid (1) | Acetic acid (2) | Hydrochloric acid (3) |
| Acid concentration (D, eq) | 1 | 2 | 3 |
| Molar Ratio (E, eq) | 0.5 (1) | 1 (2) | 2 (3) |

**Supplementary Table 2** Box–Behnken experimental design and the results for yield of derivatization of HAN- monosaccharide

| Run | A | B | C | D | E | Yield | Run | A | B | C | D | E | Yield |
| --- | --- | --- | --- | --- | --- | --- | --- | --- | --- | --- | --- | --- | --- |
| 1 | 6 | 70 | 2 | 2 | 2 | 23 | 24 | 4 | 90 | 2 | 3 | 2 | 43 |
| 2 | 4 | 80 | 2 | 1 | 1 | 43 | 25 | 4 | 90 | 2 | 2 | 1 | 43 |
| 3 | 6 | 80 | 3 | 2 | 2 | 0 | 26 | 4 | 90 | 1 | 2 | 2 | 54 |
| 4 | 4 | 80 | 2 | 3 | 3 | 49 | 27 | 4 | 80 | 2 | 2 | 1 | 62 |
| 5 | 2 | 90 | 2 | 2 | 2 | 26 | 28 | 4 | 80 | 2 | 2 | 2 | 54 |
| 6 | 2 | 70 | 2 | 2 | 2 | 23 | 29 | 4 | 80 | 3 | 3 | 2 | 0 |
| 7 | 4 | 70 | 2 | 3 | 2 | 23 | 30 | 6 | 90 | 2 | 2 | 2 | 44 |
| 8 | 2 | 80 | 3 | 2 | 2 | 0 | 31 | 4 | 70 | 2 | 2 | 3 | 23 |
| 9 | 4 | 70 | 2 | 1 | 2 | 23 | 32 | 4 | 90 | 2 | 2 | 3 | 49 |
| 10 | 4 | 90 | 3 | 2 | 2 | 0 | 33 | 4 | 80 | 1 | 2 | 3 | 49 |
| 11 | 4 | 70 | 2 | 2 | 1 | 23 | 34 | 2 | 80 | 2 | 1 | 2 | 26 |
| 12 | 4 | 80 | 2 | 2 | 2 | 54 | 35 | 4 | 70 | 3 | 2 | 2 | 0 |
| 13 | 2 | 80 | 2 | 3 | 2 | 26 | 36 | 2 | 80 | 2 | 2 | 3 | 26 |
| 14 | 4 | 80 | 2 | 1 | 3 | 49 | 37 | 4 | 80 | 2 | 3 | 1 | 53 |
| 15 | 2 | 80 | 1 | 2 | 2 | 54 | 38 | 6 | 80 | 1 | 2 | 2 | 44 |
| 16 | 6 | 80 | 2 | 1 | 2 | 44 | 39 | 4 | 80 | 1 | 3 | 2 | 53 |
| 17 | 2 | 80 | 2 | 2 | 1 | 26 | 40 | 4 | 80 | 2 | 2 | 2 | 54 |
| 18 | 6 | 80 | 2 | 2 | 1 | 44 | 41 | 4 | 90 | 2 | 1 | 2 | 43 |
| 19 | 4 | 80 | 3 | 1 | 2 | 0 | 42 | 4 | 70 | 1 | 2 | 2 | 23 |
| 20 | 4 | 80 | 2 | 2 | 2 | 54 | 43 | 4 | 80 | 1 | 1 | 2 | 54 |
| 21 | 6 | 80 | 2 | 3 | 2 | 53 | 44 | 4 | 80 | 1 | 2 | 1 | 55 |
| 22 | 4 | 80 | 2 | 2 | 2 | 54 | 45 | 6 | 80 | 2 | 2 | 3 | 44 |
| 23 | 4 | 80 | 3 | 2 | 3 | 0 | 46 | 4 | 80 | 3 | 2 | 1 | 0 |

**Supplementary Table 3** Analysis of variance of the experimental results of the BBD in derivatization of HAN-monosaccharide

| **Source** | **Sum of Squares** | **df** | **Mean Square** | **F-value** | **p-value** |
| --- | --- | --- | --- | --- | --- |
| **Model** | 16617.52 | 20 | 830.88 | 20.4 | < 0.0001 |
| A | 495.06 | 1 | 495.06 | 12.16 | 0.0018 |
| B | 1242.56 | 1 | 1242.56 | 30.52 | < 0.0001 |
| C | 9312.25 | 1 | 9312.25 | 228.69 | < 0.0001 |
| D | 20.25 | 1 | 20.25 | 0.5 | 0.4872 |
| E | 5.58 | 1 | 5.58 | 0.14 | 0.7144 |
| AB | 81 | 1 | 81 | 1.99 | 0.1707 |
| AC | 25 | 1 | 25 | 0.61 | 0.4407 |
| AD | 20.25 | 1 | 20.25 | 0.5 | 0.4872 |
| AE | 0 | 1 | 0 | 0 | 1 |
| BC | 240.25 | 1 | 240.25 | 5.9 | 0.0227 |
| BD | 0 | 1 | 0 | 0 | 1 |
| BE | 9 | 1 | 9 | 0.22 | 0.6423 |
| CD | 0.25 | 1 | 0.25 | 0.01 | 0.9382 |
| CE | 9 | 1 | 9 | 0.22 | 0.6423 |
| DE | 25 | 1 | 25 | 0.61 | 0.4407 |
| A² | 1182.78 | 1 | 1182.78 | 29.05 | < 0.0001 |
| B² | 1937.82 | 1 | 1937.82 | 47.59 | < 0.0001 |
| C² | 3913.32 | 1 | 3913.32 | 96.1 | < 0.0001 |
| D² | 236.37 | 1 | 236.37 | 5.8 | 0.0237 |
| E² | 195.19 | 1 | 195.19 | 4.79 | 0.0381 |
| **Residual** | 1017.98 | 25 | 40.72 |  |  |
| Lack of Fit | 1017.98 | 21 | 48.48 |  |  |
| Pure Error | 0 | 4 | 0 |  |  |
| **R^2^=0.9423** | R^2^_Adj_=0.8961 | R^2^_Pred_=0.7899 | CV=18.50% | Adeq Precision=16.1292 | |
